# The application of rapid response team in category 1 emergency caesarean section teaching for OBGYN residents in the delivery room

**DOI:** 10.1097/MD.0000000000034551

**Published:** 2023-09-01

**Authors:** Xia Xu, Ying Lin, Ling Weng, Yanni Guo, Lin Lin, Jianying Yan

**Affiliations:** a College of Clinical Medicine for Obstetrics and Gynecology and Pediatrics, Fujian Medical University, Fuzhou, Fujian Province, China; b Department of Obstetrics and Gynecology, Fujian Maternity and Child Health Hospital, Fuzhou, Fujian Province, China.

**Keywords:** delivery room, emergency cesarean section, rapid response team, resident training

## Abstract

Category 1 cesarean section (CS) can be a life-saving procedure when there is immediate threat to the life of the woman or fetus. However, category 1 CS is a challenge for obstetrics and gynecology residents, and it is necessary to establish an effective and straightforward teaching strategy. This study aimed to evaluate the efficiency of rapid response team (RRT) on category 1 CS teaching for obstetrics and gynecology residents in the delivery room. A total of 142 residents who underwent standardized residency training programs in the delivery room were divided into a RRT teaching group and a traditional response (TR) teaching group. In the RRT teaching group, Category 1 emergency CS teaching was started and explored by rapid response team. The training included both theoretical and practical components. After the training, decision-to-delivery interval (DDI), neonatal Apgar score, operation time and rate of postpartum hemorrhage were compared. A questionnaire on the subjective assessment of various aspects of the program was conducted at the end of the training period. The DDI in minutes in the RRT teaching group (n = 72) was significantly shorter than that of the TR teaching group (n = 70) (11.83 ± 4.16 vs 13.56 ± 5.47, *P* = .0364). The score of satisfaction from residents in the RRT teaching group was significantly higher than that of the TR group [7 (6, 9) vs 9 (7, 10), *P* = .0154]. Compared with the TR teaching group, more residents thought their clinical skills have been improved (94.29% vs 100%, *P* = .0396) and willing to recommend their training method to others (91.43% vs 100%, *P* = .0399) in the RRT teaching group. However, no significant differences were observed in the incidence of postpartum hemorrhage between the 2 groups. RRT teaching is beneficial in the standardized training and teaching of residents in the delivery room. It improves the DDI of category 1 emergency cesarean section and the degree of satisfaction.

## 1. Introduction

Maternal and fetal morbidity and mortality are significant public health concerns in worldwide.^[1–5]^ Obstetric emergencies in the delivery room such as uterine rupture, placenta abruption and cord prolapse are significant contributors.^[6–9]^ Emergency cesarean section (CS) sometimes is a life-saving procedure for these life-threatening condition.^[10,11]^ Depending on the severity of the fetal and/or maternal condition, emergency CS is categorized into 4-category^[12]^ that are category 1, category 2, category 3 and category 4. Category 1 CS is when there is immediate threat to the life of the woman or fetus, for example, suspected uterine rupture, major placental abruption, cord prolapse, fetal hypoxia or persistent fetal bradycardia. Team performance, rapid response, effective communication, critical thinking, and leadership are recommended during Category 1 CS to improve perinatal outcomes. Rapid response team (RRT) is a multidisciplinary team staffed by healthcare professionals with expertise in critical care. RRT was established and applied in Category 1 CS in Fujian Maternity and Child Health Hospital since 2015. Not surprisingly, the application of RRT improved perinatal outcomes as reported in other studies.

Resident training is a crucial stage of progressing for a obstetrics and gynecology doctor. During this procedure, resident doctors need to preview and accept a large amount of theoretical knowledge, practice and command basic clinical skills and learn to handle kinds of severe emergency situation. Residents are important members of the delivery team and should be prepared and trained against all risks including Category 1 CS in the delivery room.

However, whether RRT can be quickly mastered by residents and improve the perinatal outcomes remains to be elucidated. Therefore, this study aimed to study the perinatal outcomes of patients and explore the self-evaluation and satisfaction of residents after receiving RRT training.

## 2. Methods

### 2.1. Study population

**Figure 1.**
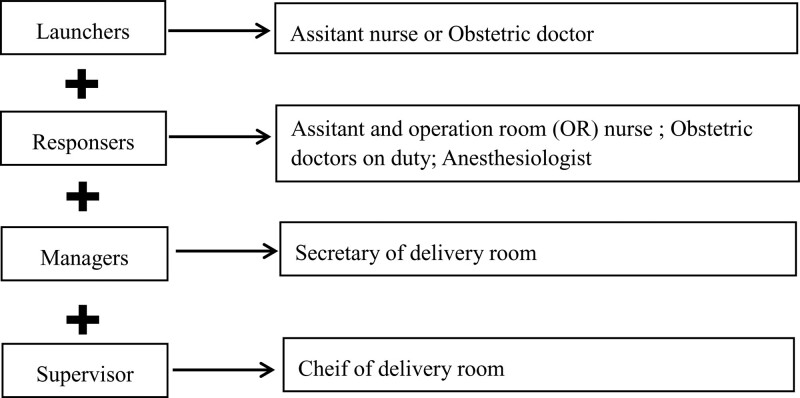
Composition of rapid response team for emergency cesarean section.

**Figure 2.**
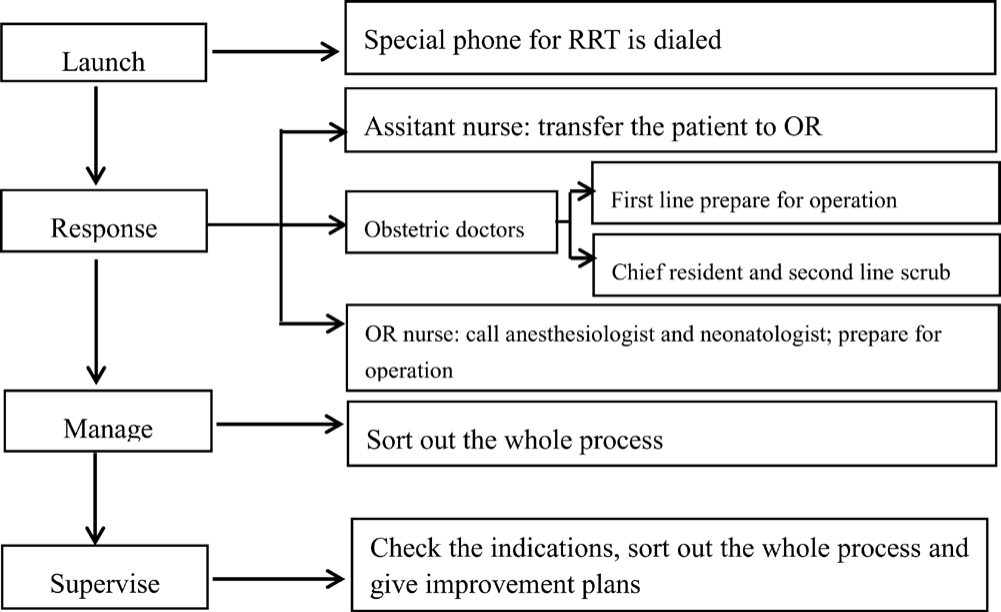
Schematic diagram of rapid response team for emergency cesarean section.

All residents who underwent resident standardizing training from January 2020 to January 2021 in the delivery room of Fujian Maternity and Child Health Hospital accepted traditional response (TR) teaching (n = 70) and all residents who underwent resident standardizing training from January 2021 to January 2022 in the delivery room of Fujian Maternity and Child Health Hospital accepted RRT teaching (n = 72). The theory and program of emergency CS especially Category 1 were introduced to all residents in the first week of training. Residents in TR teaching group practiced response to category 1 CS in traditional response mode that category 1 CS was launched and responded by resident-self and managed by teacher. Residents in RRT teaching group practiced response to category 1 CS in RRT mode. The composition and schematic diagram of RRT was showed in Figure 1 and Figure 2. It is a case control study, so there was no written informed consent for patient in this study. All the procedures were followed in accordance with Declaration of Helsinki. The ethics committee of Fujian Maternity and Child Health Hospital approved this consent process and also specifically approved this study (Grant number: 2022KYLLRK1103).

Category 1 emergency CS was launched once immediate threat to the life of the woman or fetus (for example, suspected uterine rupture, major placental abruption, cord prolapse, fetal hypoxia or persistent fetal bradycardia). RRT was applied in category 1 CS in Fujian Maternity and Child Health Hospital since 2015. The policy and process of RRT had been established and refined.

### 2.2. Outcome measures

Baseline characteristics of all residents enrolled in the study was analyzed and compared and achieved satisfactory teaching results, which can provide a basis for promoting the clinical training teaching method of category 1 CS in the future, which is reported as follows. The outcomes of all patients who underwent category 1 CS from January 2020 to January 2021 in the delivery room of Fujian Maternity and Child Health Hospital was compared with that of all patients who underwent category 1 CS from January 2021 to January 2022.

## 3. Statistical analysis

The normality of data was assessed by the Kolmogorov–Smirnov test. The categorical variables are expressed in percentages, the continuous variables with normal distribution are expressed as mean ± SD, and the nonnormally distributed continuous variables are expressed as median (quartile spacing) values. For 2 groups with continuous variables, the 2-sample independent t-test was used when each set of data was normally distributed with homogeneous variance; otherwise, the Mann–Whitney *U* test was used. Categorical variables were analyzed with the Chi-square test or Fisher exact probability method. All statistical analyses were performed using the Statistical Package for the Social Sciences for Windows v22.0 (SPSS Inc., Chicago, IL). Differences were considered statistically significant if *P* < .05.

## 4. Results

There were 70 residents in the TR group and 72 residents in the RRT group. There were no significant differences in age, gender, education and entrance examination scores between the 2 groups (Table 1).

**Table 1:** Baseline characteristics of all residents enrolled in the study.

Parameters	TR-G (n = 70)	RRT-G (n = 72)	*P* value
Age (mean ± SD, yr)	26.72 ± 4.04	25.43 ± 3.72	.051
Gender (No. [%])			.585
Male	13	16	
Female	57	56	
Education (No. [%])			.310
Undergraduate	17	23	
Postgraduate or doctor	53	49	
Entrance examination scores	83.43 ± 6.72	82.72 ± 7.64	.555

G = group, RRT = rapid response team, TR = traditional response.

After the training, there were no apparent differences in the baseline characteristics of the patients (age, dilatation of cervix, and history of cesarean section) that we included between the 2 groups (Table 2). The decision-to-delivery internal (DDI) in minutes in the RRT teaching group was significantly shorter than that of the TR teaching group (11.83 ± 4.16 vs 13.56 ± 5.47, *P* = .0364). However, no significant differences were observed in the neonatal Apgar score, weight of newborn and incidence of postpartum hemorrhage (PPH) between the 2 groups.

**Table 2:** Comparison of clinical characteristics and outcomes between the two groups.

Characteristics	TR-G (n = 70)	RRT-G (n = 72)	*P* value
Maternal age (mean ± SD, yr)	28.52 ± 5.68	29.27 ± 6.41	.464
Dilatation of cervix (mean ± SD, cm)	5.35 ± 1.12	6.53 ± 1.42	1.711
History of cesarean section			.672
None	68	69	
One	2	3	
DDI (mean ± SD, yr)	13.56 ± 5.47	11.83 ± 4.16	.0364
Neonatal Apgar score (median [IQR], score)	8 (7, 9)	9 (7, 10)	.354
Neonatal gender (No. [%])			.729
Boy	32	35	
Girl	38	37	
Weight of nenborn (median [IQR], g)	3450.77 ± 17.98	3368.34 ± 16.29	2.111
Postpartum hemorrhage (No. [%])	3 (4.29)	4 (5.56)	.727

DDI = decision-to-delivery interval, G = group, PPH = postpartum hemorrhage, RRT = rapid response team, TR = traditional response.

A total of 142 questionnaires were distributed in 2 groups and 142 were recovered, representing a 100% return rate. Before data entry, all questionnaires were checked for logic and integrity. As shown in Table 3, the score of satisfaction from residents in the RRT teaching group was significantly higher than that of the TR group [7 (6, 9) vs 9 (7, 10), *P* = .0154]. Compared with the TR teaching group, more residents thought their clinical skills have been improved (94.29% vs 100%, *P* = .0396) and willing to recommend their training method to others (91.43% vs 100%, *P* = .0399) in the RRT teaching group. However, no significant differences were observed in the incidence of PPH between the 2 groups.

**Table 3:** Comparison of the results of questionnaire between the two groups.

Entries	TR-G (n = 70)			RRT-G (n = 72)			*P* value
	Yes	No	Uncertain	Yes	No	Uncertain	
Have you improved your learning interests?	64	6	0	68	4	0	.483
Have you improved your clinical skills?	66	0	4	72	0	0	.0396
Have you improved your theoretical knowledge of emergency caesarean section?	69	0	1	72	0	0	.309
Will you recommend this method to others?	64	4	2	72	0	0	.0399
Is it helpful for your clinical work in the future?	65	0	5	69	0	3	.442
Teaching satisfaction score (median [IQR])	7 (6, 9)			9 (7, 10)			.0154

G = group, RRT = rapid response team, TR = traditional response.

## 5. Discussions

Urgent situation including suspected uterine rupture, major placental abruption, cord prolapse, fetal hypoxia or persistent fetal bradycardia sometimes occurs in the delivery room. Category 1 CS can be a life-saving procedure for ABOVED life-threaten emergencies. RRT was applied in category 1 CS in Fujian Maternity and Child Health Hospital since 2015. Residents are important members of the delivery team and should be prepared against Category 1 CS in the delivery room. However, resident routine training in delivery room was in traditional response mode. Whether RRT teaching mode can be quickly mastered by residents and improve the perinatal outcomes remains unclear. Therefore, this study aimed to study the perinatal outcomes of patients and explore the self-evaluation and satisfaction of residents after application of RRT teaching in residents about Category 1 CS. The primary finding was that DDI in minutes in the RRT teaching group was shorter than that of the TR teaching group and no increase in complications. We also found that more residents thought their clinical skills had been improved, satisfy and willing to recommend RRT training method to others. However, no significant differences were observed in the incidence of PPH between the 2 groups.

CS is the commonest major obstetric surgery and a life-saving intervention sometimes. It is divided into planed CS and emergency CS.^[13]^ Take into account the condition of the woman and the unborn baby, emergency CS is categorized into 4-category. Category 1- Immediate threat to the life of the woman or fetus. Category 2 - Maternal or fetal compromise which is not immediately life-threatening. Category 3 - No maternal or fetal compromise but needs early birth. Category 4 -Birth timed to suit woman or healthcare provider. To improve maternal and neonatal outcomes, it is recommended that the time from decision of category 1 emergency CS to the birth of the baby (decision-to-delivery interval, DDI) should be within 30 minutes.^[9,14,15]^ In order to achieve the 30 minutes goal, the whole process of category 1 CS that should be performed perfectly as far as possible. Accordingly, a rapid response multidisciplinary team, including, obstetricians, anesthesiologist, pediatricians, and other related personnel was established in order to shorten the DDI for category 1 emergency CS. As some studies reported, 30 minutes goal can be achieved in this study after application of RRT.

However, It should be noted that category 1 emergency CS is launched against with immediate threat to life of the woman or fetus. Some studies demonstrated that short DDI could improves perinatal outcomes. Our hypothesis was that resident trainees act as front-line caregivers in delivery room and an important member during category 1 emergency CS, accept RRT teaching may short DDI. The DDI in the RRT teaching group indeed shorter. The results reflect that strengthen collaboration of residents with all related staff can short DDI. However, the incidence of PPH did not increase. This was similar to other previous studies which demonstrated that various quality improvement programs including continuous education and team training course for obstetric and related staff with emphasis on the importance of achieving the standard of 30 minutes goal, can significantly shorten DDI and other processes.^[16–18]^

This study has some limitations. First, to ensure the homogeneity of educational interventions, these data were obtained at a single teaching hospital, and validation from other institutions is needed. Second, RRT need practice again and again. So RRT teaching is time-consuming. Most resident trained in the delivery room for 2 months. Undoubtedly, this may reduce the effect of RRT training. Third, this is a case control study and if a randomized controlled trial can be performed will be better.

## 6. Conclusion

The application of RRT teaching in the standardized teaching of residents improves the DDI of category 1 emergency cesarean section and the degree of satisfaction. We therefore recommend this teaching method.

## Author contributions

**Conceptualization:** Xia Xu, Ying Lin, Ling Weng, Yanni Guo, Lin Lin, Jianying Yan.

**Data curation:** Xia Xu, Ying Lin, Ling Weng, Yanni Guo, Lin Lin, Jianying Yan.

**Formal analysis:** Xia Xu, Ying Lin, Ling Weng, Yanni Guo, Lin Lin, Jianying Yan.

**Funding acquisition:** Jianying Yan.

**Investigation:** Xia Xu, Ying Lin, Ling Weng, Yanni Guo, Lin Lin, Jianying Yan.

**Methodology:** Xia Xu, Ying Lin, Ling Weng, Yanni Guo, Lin Lin, Jianying Yan.

**Project administration:** Xia Xu, Ying Lin, Ling Weng, Yanni Guo, Lin Lin, Jianying Yan.

**Resources:** Xia Xu, Ying Lin, Ling Weng, Yanni Guo, Lin Lin, Jianying Yan.

**Software:** Xia Xu, Ying Lin, Ling Weng, Yanni Guo, Lin Lin, Jianying Yan.

**Supervision:** Xia Xu, Ying Lin, Ling Weng, Yanni Guo, Lin Lin, Jianying Yan.

**Validation:** Xia Xu, Ying Lin, Ling Weng, Yanni Guo, Lin Lin, Jianying Yan.

**Visualization:** Xia Xu, Ying Lin, Ling Weng, Yanni Guo, Lin Lin, Jianying Yan.

**Writing – original draft:** Xia Xu, Ying Lin, Ling Weng, Yanni Guo, Lin Lin, Jianying Yan.

**Writing – review & editing:** Xia Xu, Ying Lin, Ling Weng, Yanni Guo, Lin Lin, Jianying Yan.
